# GDSL Lipase Gene *HTA1* Negatively Regulates Heat Tolerance in Rice Seedlings by Regulating Reactive Oxygen Species Accumulation

**DOI:** 10.3390/antiox13050592

**Published:** 2024-05-11

**Authors:** Rui Su, Jingkai Luo, Yingfeng Wang, Yunhua Xiao, Xiong Liu, Huabing Deng, Xuedan Lu, Qiuhong Chen, Guihua Chen, Wenbang Tang, Guilian Zhang

**Affiliations:** 1 College of Agronomy, Hunan Agricultural University, Changsha 410000, China; surui0605@stu.hunau.edu.cn (R.S.); lomulo@stu.hunau.edu.cn (J.L.); wangyf@stu.hunau.edu.cn (Y.W.); yhxiao@hunau.edu.cn (Y.X.); xiongliu@whu.edu.cn (X.L.); denghuabing@hunau.edu.cn (H.D.); luxuedan@hunau.edu.cn (X.L.); cqh924@hunau.edu.cn (Q.C.); chen_gh@hunau.edu.cn (G.C.); 2 Hunan Provincial Key Laboratory of Rice and Rapeseed Breeding for Disease Resistance, Changsha 410000, China; 3 Hunan Hybrid Rice Research Center, Hunan Academy of Agricultural Sciences, Changsha 410000, China; 4 State Key Laboratory of Hybrid Rice, Changsha 410000, China

**Keywords:** rice, heat-responsive genes, reactive oxygen species (ROS), GDSL lipase

## Abstract

High temperature is a significant environmental stress that limits plant growth and agricultural productivity. GDSL lipase is a hydrolytic enzyme with a conserved GDSL sequence at the N-terminus, which has various biological functions, such as participating in plant growth, development, lipid metabolism, and stress resistance. However, little is known about the function of the GDSL lipase gene in the heat tolerance of rice. Here, we characterized a lipase family protein coding gene *HTA1*, which was significantly induced by high temperature in rice. Rice seedlings in which the mutant *hta1* was knocked out showed enhanced heat tolerance, whereas the overexpressing *HTA1* showed more sensitivity to heat stress. Under heat stress, *hta1* could reduce plant membrane damage and reactive oxygen species (ROS) levels and elevate the activity of antioxidant enzymes. Moreover, real-time quantitative PCR (RT-qPCR) analysis showed that mutant *hta1* significantly activated gene expression in antioxidant enzymes, heat response, and defense. In conclusion, our results suggest that *HTA1* negatively regulates heat stress tolerance by modulating the ROS accumulation and the expression of heat-responsive and defense-related genes in rice seedlings. This research will provide a valuable resource for utilizing *HTA1* to improve crop heat tolerance.

## 1. Introduction

Rice, the most important global food crop, feeds nearly two-thirds of the world’s population and provides over 21% of the world’s energy intake and 76% of the energy intake in Southeast Asia [1]. In China, the annual cultivated area of rice is about 30 million hectares, contributing to 40% of the country’s total grain output [2]. However, in recent years, due to factors such as the greenhouse effect, the global climate has been warming year by year, and some rice-growing regions often experience persistent extreme high temperatures [3]. The global temperature is increasing year by year, and the IPCC report predicts that by 2100, the global average temperature in Southeast Asia will increase by 3–5 °C [4]. For every 1 °C increase in temperature, rice production will decrease by 3.2% [5]. This heat damage seriously impairs the normal growth and development of rice, affecting key processes such as respiration, transpiration, organ formation, photosynthesis, and enzyme activity [6]. It poses a significant threat to rice production and thus has a serious impact on global food security.

The optimal growth temperature for rice seedlings is 25–28 °C. High temperatures above 32 °C can cause some negative effects on all stages of rice growth and development. Exposure to temperatures ranging from 42 to 45 °C during the seedling stage can lead to increased water loss, wilting and yellowing of leaves, impeded growth of seedlings and roots, and even death of seedlings [7,8,9]. Rice seedlings at different high temperatures employ different strategies to withstand heat stress. The higher the temperature, the more protective mechanisms are activated by the seedlings. When rice seedlings are subjected to intense heat stress at 45 °C, HSP-related protective mechanisms are induced in addition to the photosynthetic protection and antioxidant pathways that are induced during mild (35 °C) or moderate (above 40 °C) heat stress [10].

GDSL esterases are enzymes with important functions that are widely found in organisms. The special feature of this enzyme is its N-terminal conserved GDSL sequence, containing a conserved Gx, Sx, G motif and four conserved domains. It can be classified into the SGNH subfamily, and its flexible catalytic site distinguishes it from other hydrolases while also leading to structural and functional diversity among family members. Therefore, this enzyme has a wide range of substrate specificities and can hydrolyze various substrates, including thioesters, phosphates, and aromatic esters [11,12]. GDSL lipases play a role in plant nutrient growth, reproductive growth, and plant metabolism, while increasing evidence suggests that they are involved in plant biotic or abiotic stress responses [13]. In rice, GDSL esterases constitute a sizable gene family with 115 members [14]. For example, *OsGELP34*, encoded by a GDSL esterase family member in rice, is mainly expressed during pollen wall formation. *GER1* and *WDLI* regulate rice coleoptile growth and seedling growth [15]. In *Arabidopsis thaliana*, *CEDF1*, which encodes a GDSL esterase, degrades cuticular wax on the stigma surface, promoting pollen tube development [16]. Tomato GDSL1 is involved in cuticular wax accumulation outside cells, reducing fruit cuticle thickness [17]. Other studies have also found that the expression of GDSL esterase-related genes can be induced by abiotic stress factors [18], indicating that these enzymes participate in stress responses.

Although some scholars have studied the important role of GDSL esterases in physiological processes such as plant stress responses and secondary metabolism and have identified a few family members [19,20], the biological functions of most individual genes encoding this enzyme are still poorly understood. Unfortunately, currently, there is no report on the response of GDSL esterases to crop heat stress. Here, we investigated the response mechanism of *HTA1*, a gene encoding GDSL esterases, to heat stress in rice plants using overexpression and gene editing of gene-edited mutant rice lines.

## 2. Materials and Methods

### 2.1. Plant Materials

In this study, the japonica rice variety Zhonghua 11 (ZH11) collected at Hunan Agricultural University was used as the wild type (WT). The coding sequence (CDS) of *HTA1* was cloned using ZH11 cDNA as template and inserted into the pCAMBIA1300 vector. To generate *HTA1* knockout mutants, sgRNA was designed using the website tool CRISPRdirect [21] and constructed by simultaneous digestion and ligation into pBWA(V)HU-ylcas9. The constructed recombinant binary vector was then transformed into rice callus by *Agrobacterium*-mediated transformation using EHA105. Further experiments were carried out by screening positive plants for resistance to Hygromycin B (31282-04-9, Roche, Shanghai, China) [22]. Transcript levels of the overexpression lines were detected using real-time quantitative PCR. Detection of mutation types in mutant lines was performed using PCR. OE-1 and OE-2 and *hta1-1* and *hta1-2* were used for further experiments.

### 2.2. Heat Stress Treatment

Seeds of the wild type (ZH11), *HTA1* overexpressing lines (OE-1 and OE-2), and mutants (*hta1-1* and *hta1-2*) were germinated for 48 h at 37 ± 1 °C in distilled water. They were then seeded in bottomless 96-well plates and transferred to a culture chamber. For the first two days, seedlings were grown in distilled water. From the third day, the water was changed into Kimura B nutrient solution with a concentration of pH 5.6 to 5.8 [23]. The nutrient solution was changed every two days and the seedlings were grown in the culture chamber for 10 days. The incubation conditions were: temperature 25 °C, light intensity 30,000 lux, 12 h of day/night and relative humidity 70 ± 7%. Subsequently, the *HTA1* overexpression and mutant lines were transferred to a 45 °C light incubator for 32 and 38 h, respectively. Seedlings at the end of the heat treatment were recovered at 25 °C for 7 d. Control-treated plants were grown at 25 °C at all times. At the end of recovery, phenology and survival of treated and control plants were recorded. Each treatment had four biological replicates with 30 seedlings per replicate.

### 2.3. Determination of Reactive Oxygen Species and Antioxidant Enzyme Activities

At the end of the heat treatment, the inverted second leaves of seedlings from each treatment were sampled. The relative electrolyte leakage rate, malondialdehyde (MDA) content, antioxidant enzyme activities, and ROS accumulation were determined for WT and transgenic lines after control and heat treatments using methods as described previously [24,25]. Measurement of the relative electrolyte leakage rate was performed with the conductivity meter (DS-11A). Measurement of MDA content was performed by the thiobarbituric acid (TBA) colorimetric method. SOD and POD activities were determined using the riboflavin nitro blue tetrazolium (NBT) method and the guaiacol method, respectively. Leaves were stained using 3,3-diaminobenzidine (DAB) and NBT as described previously [26]. H_2_O_2_ and O_2_^•−^ content of each treated plant was determined using a kit (BC3595, Solarbio, Shanghai, China).

### 2.4. Quantitative Real-Time PCR Analysis

Leaves of ZH11 plants treated with heat at 45 °C for 0 h, 1.5 h, 3 h, 6 h, 12 h, and 24 h, as well as leaves of plants heat-treated for 32 h and control-treated WT and transgenic lines, were collected and the corresponding RNAs were extracted for detection of transcript levels of the relevant genes. Total RNA was extracted according to the kit method (R701-01, Vazyme, Nanjing, China). Reverse transcription was performed using a kit (R223-01, Vazyme). According to the steps of the kit, 4× gDNA wiper Mix was used to remove the residual genomic DNA, and then the gDNA wiper action was terminated and the reverse transcription reaction was performed. Rice *OsActin1* was used as an internal reference gene. Relative changes of gene expression levels were quantified by the 2^−ΔΔCT^ method [27]. The primer sequences used for RT-qPCR are shown in Supplementary Materials Table S1.

### 2.5. Statistical Analysis

Data were analyzed by one-way ANOVA using DPS (version 7.05) and plotted using GraphPad Prism (version 8.01).

## 3. Results

### 3.1. Transcript Profiles of Rice Seedling Stage HTA1 to Heat Stress

**Figure 1:**
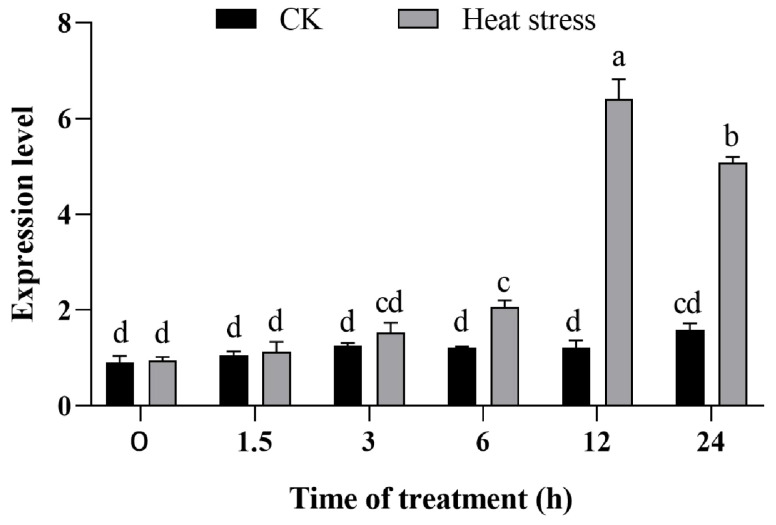
Expression levels of *HTA1* in ZH11 under heat stress. Data are mean ± standard error (*n* = 3; different letters indicate significant differences in expression levels between lines, *p* < 0.05).

In our previous study, the gene expression differences between heat-resistant variety 996 and heat-sensitive variety 4628 in anthers at the anthesis stage under heat stress were analyzed by transcriptome sequencing technology. They identified a lipase family protein-encoding gene, *HTA1*, significantly induced by heat stress. To investigate whether the transcript level of *HTA1* was affected by heat stress, we examined the expression level of *HTA1* in ZH11 seedlings after 0 h, 1.5 h, 3 h, 6 h, 12 h, and 24 h of treatment at 45 °C by RT-qPCR. Upon heat stress, the expression of *HTA1* significantly increased after 6 h and peaked at 12 h (Figure 1). These results indicated that the transcript levels of *HTA1* are responsive to heat stress and are strongly induced by heat.

### 3.2. Response of HTA1 Transgenic Seedlings to Heat Stress

**Figure 2:**
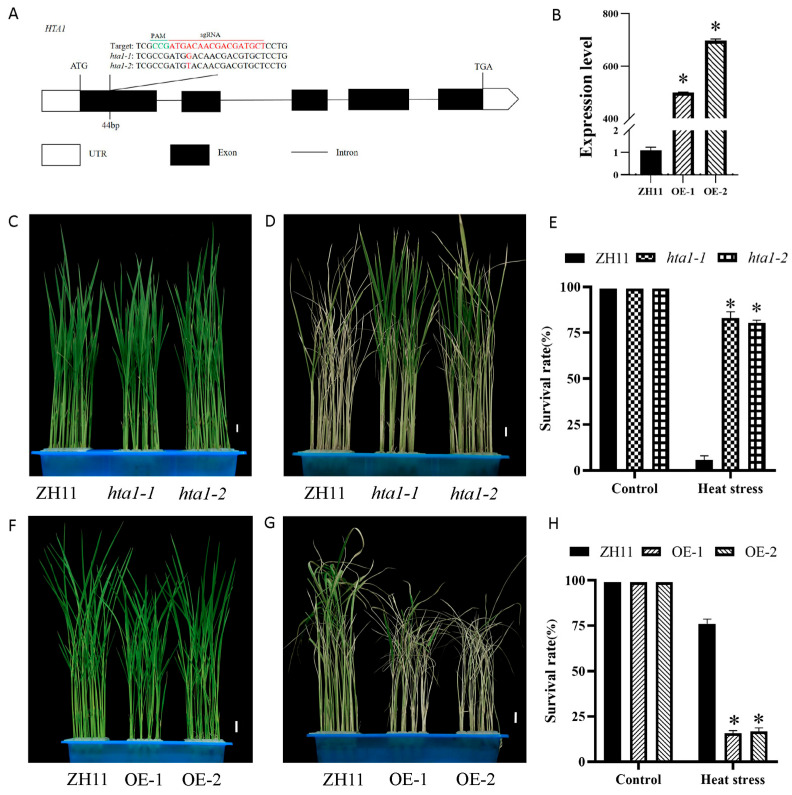
Phenotypic analyses of *HAT1* transgenic rice lines under heat stress. (**A**) Schematic representation of the CRISPR/Cas9 target site and the base mutation sequences of *hta1-1* and *hta1-2*. (**B**) Real-time fluorescent quantitative PCR analysis of *HTA1* in the WT and overexpressed lines OE-1 and OE-2. (**C**) Control groups of the WT and *hta1-1* and *hta1-2* lines. (**D**) WT and *hta1-1* and *hta1-2* lines after 32 h of heat stress followed by 7 days of recovery at optimal temperature. (**E**) Survival rates of the control group and the WT, *hta1-1*, and *hta1-2* lines after 7 days of recovery following heat treatment. (**F**) Control groups of the WT and overexpressed lines OE-1 and OE-2. (**G**) WT and OE-1 and OE-2 lines after 38 h of heat stress followed by 7 days of recovery at optimal temperature. (**H**) Survival rates of the control group and the WT, OE-1, and OE-2 lines after 7 days of recovery following heat treatment. All figures represent target values of 1 cm, with data expressed as mean ± standard deviation (*n* = 3; * p < 0.05).

To investigate the role of *HTA1* in rice under heat stress, we constructed *HTA1* mutants and overexpression lines in the background of ZH11. *hta1-1* and *hta1-2* have two different types of mutation loci at the 44 bp position of the *HTA1* exon, which, respectively, add T and G, leading to code shift mutations (Figure 2A). The expression levels of *HTA1* in OE-1 and OE-2 were significantly higher than those of ZH11 (496-fold and 581-fold, respectively) (Figure 2B). Under heat stress, the survival rates of the two mutants (*hta1-1* and *hta1-2*) reached 83% and 80%, which was significantly higher compared to the WT (Figure 2D,E). In contrast, the survival rates of the two overexpression lines (OE-1 and OE-2) were only 15.6% and 16.6% (Figure 2G,H). Under normal temperature treatment, the survival rate of all seedlings was 100% (Figure 2C,E,F,H). The results showed that *HTA1* negatively regulated the heat stress of rice seedlings.

### 3.3. Effects of Heat Stress on Membrane Lipid Peroxidation and Antioxidant Enzyme Activities of HTA1 Transgenic Seedlings

**Figure 3:**
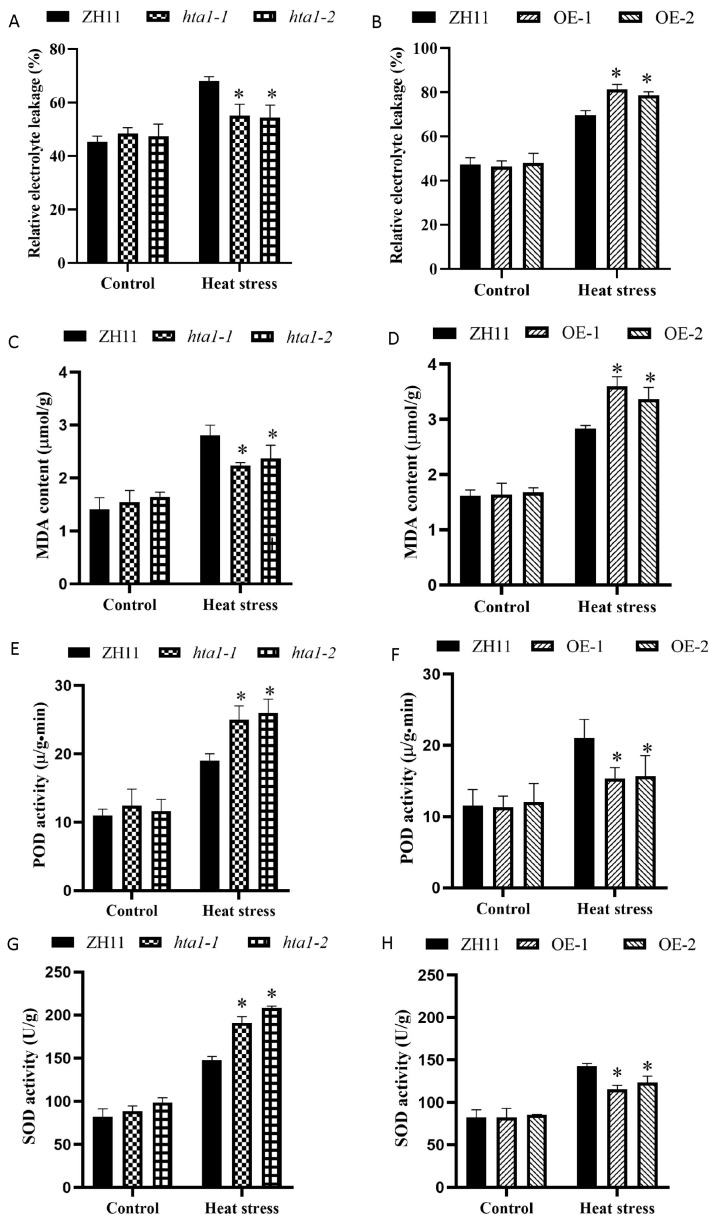
Membrane peroxidation and antioxidant enzyme activities in the WT and *HTA1* transgenic rice lines under heat stress. Relative electrolyte leakage rate of *hat1-1* and *hat1-2* (**A**) and OE-1 and OE-2 (**B**) in control and heat stress conditions. Malondialdehyde (MDA) content of *hat1-1* and *hat1-2* (**C**) and OE-1 and OE-2 (**D**) in control and heat stress conditions. Peroxidase (POD) activity of *hat1-1* and *hat1-2* (**E**) and OE-1 and OE-2 (**F**) in control and heat stress conditions. Superoxide dismutase (SOD) activity of *hat1-1* and *hat1-2* (**G**) and OE-1 and OE-2 (**H**) in control and heat stress conditions. All data are presented as mean ± standard deviation (*n* = 3; * p < 0.05).

To clarify the possible physiological mechanism by which *HTA1* affects thermotolerance in rice, we determined the relative electrolyte leakage rate, MDA content, and antioxidant enzyme activities (SOD and POD) in WT and transgenic seedlings were measured under heat stress and the relative electrolyte leakage rate was measured as a parameter reflecting plant cell membrane permeability under abiotic stress [28]. Under control conditions, there were no significant differences in relative electrolyte leakage rate and MDA content between WT and transgenic seedlings (Figure 3A,C). Under heat stress, the relative electrolyte leakage rate and MDA content in both WT and transgenic seedlings increased, whereas the increase magnitude of the relative electrolyte leakage rate and MDA content of *hta1* were significantly lower than that of the WT (Figure 3A,C). Under heat stress, the relative electrolyte leakage rate and MDA content of the overexpression lines showed the opposite trend of Figure 3B,D. These results indicated that *HTA1* mutants can reduce membrane damage in rice seedlings under heat stress.

Next, we measured antioxidant enzyme activities, including SOD and POD, in WT and transgenic lines. The results showed that there were no significant differences in antioxidant enzyme activities in WT and transgenic lines under 25 °C treatment. Under heat stress, the antioxidant enzyme activities of *hta1* were significantly higher than those of the WT (Figure 3E,G), while the antioxidant enzyme activities of the overexpression lines were significantly lower than that of the WT (Figure 3F,H). These results indicate that the *HTA1* mutant could improve the heat stress tolerance of rice seedlings by increasing the antioxidant enzyme activities, thereby reducing membrane damage.

### 3.4. Effect of Heat Stress on the Reactive Oxygen Species Levels of HTA1 Transgenic Seedlings

**Figure 4:**
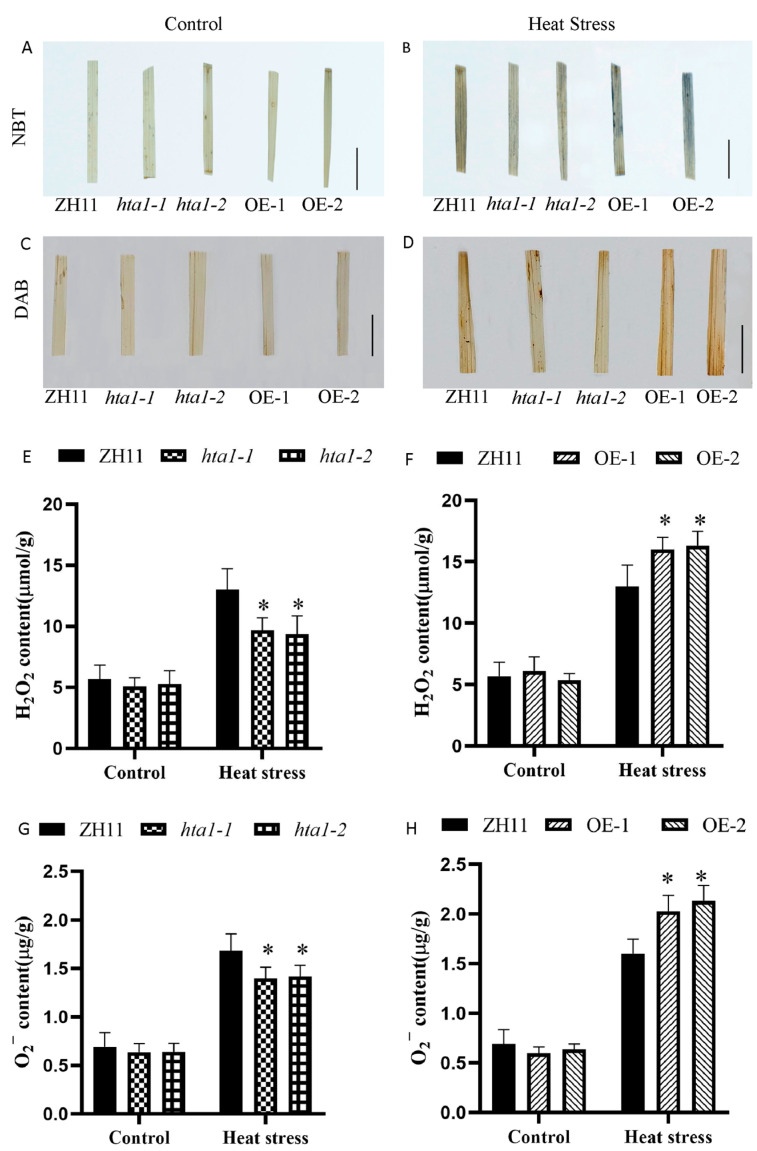
Hydrogen peroxide and superoxide anion levels in the WT and *HTA1* transgenic rice lines under heat stress. Under control and heat stress conditions, the leaves of *hat1* and overexpressed plants were stained with NBT (**A**,**B**) and DAB (**C**,**D**). (**E**,**F**) Under control and heat stress conditions, the hydrogen peroxide content of the *hta1* and overexpressed plants. (**G**,**H**) Under control and heat stress conditions, the superoxide anion content of the *hta1* and overexpressed plants. The target values in the figure are all 1 cm. The data are presented as mean ± standard deviation (*n* = 3; * p < 0.05).

Due to the significant increase in antioxidant enzyme activity in the mutant lines under heat treatment, this prompted us to assay for ROS accumulation. Under control conditions, DAB and NBT staining showed that the leaves of transgenic seedlings were not significantly different from those of the WT seedlings (Figure 4A,C). Under heat stress, the leaves of the overexpression line OE-1 and OE-2 seedlings stained darker than those of the WT, and the leaves of the *hta1* seedlings stained lighter than those of the WT (Figure 4A,B). Further, the determination result of the ROS accumulation for *HTA1* transgenic and WT seedlings showed that overexpression lines OE-1 and OE-2 accumulated significantly more H_2_O_2_ and O_2_^•−^ than the WT under heat stress, the H_2_O_2_ content in overexpression lines OE-1 and OE-2 was 23.07% and 25.63% higher than that of the WT, respectively (Figure 4F), and the O_2_^•−^ content in overexpression lines OE-1 and OE-2 was 20.80% and 26.78% higher than that of the WT, respectively (Figure 4H). In addition, the accumulation of H_2_O_2_ and O_2_^•−^ in the mutants *hta1-1* and *hta1-2* was significantly lower than that of the WT under heat stress, the H_2_O_2_ content in the mutants *hta1-1* and *hta1-2* was 34.29% and 39.3% lower than that of the WT, respectively (Figure 4E), and the O_2_^•−^ content in the mutants *hta1-1* and *hta1-2* was 20.86% and 18.3% lower than that of the WT, respectively (Figure 4G). These results, combined with changes in antioxidant enzyme activities, indicated that *HTA1* negatively regulates heat tolerance in rice seedlings by modulating antioxidant enzyme activities and thus ROS accumulation.

### 3.5. Transcriptional Changes of Antioxidant- and Defense-Related Genes in HTA1 Transgenic Seedlings under Heat Stress

To clarify the potential molecular mechanism by which *HTA1* regulates heat tolerance in rice seedlings, we first examined the transcript levels of antioxidant- and defense-related genes in the leaves of each treated plant. These genes included three antioxidant- related genes (*OsCATB* encoding catalase, *Fe^+^SOD* encoding superoxide dismutase, and *OsAXP1* encoding ascorbate peroxidase) [29,30,31] and three defense-related genes (*OsSNAC1* encoding NAC transcription factor gene, *OsDREB2A* encoding EREBP transcription factor, and *OsLEA3* encoding late embryogenesis abundant enriched protein) [32,33,34].

**Figure 5:**
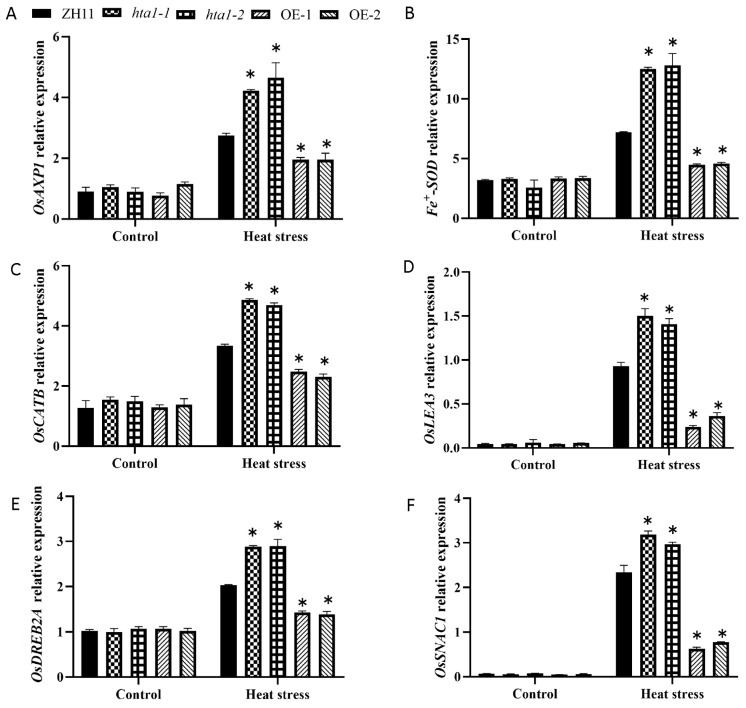
Transcriptional expression of antioxidant- (**A**–**C**) and defense-related (**D**–**F**) genes in WT, *hat1-1*, *hat1-2*, OE-1, and OE-2 plants under control and heat stress. The data are presented as mean ± standard deviation (*n* = 3; * p < 0.05).

The results showed that no significant differences in the expression levels of antioxidant genes were observed among the treated seedlings under control conditions. The expression of antioxidant genes in both WT and *HTA1* transgenic seedlings were up-regulated under heat stress conditions, especially *Fe^+^SOD*. Compared with the WT, the expression levels of the three antioxidant-related genes were significantly higher in the *hta1* under heat stress, while the expression levels of antioxidant-related genes were significantly lower in the *HTA1* overexpression lines (Figure 5A–C). Subsequently, the expression levels of the three defense genes in the WT and *HTA1* transgenic lines were measured under heat stress conditions. Under control conditions, the expression levels of defense-related genes in WT and *HTA1* transgenic seedlings were not significantly different. Under heat stress conditions, the expression levels of the three defense-related genes in the *hta1* were significantly higher than that of WT, with *OsLEA3* showing the most significant increase. The expression levels of defense-related genes in the OE-1 and OE-2 were significantly lower than those of WT (Figure 5D–F).

### 3.6. Transcriptional Changes in Genes Related to Heat Responsiveness in HTA1 Transgenic Seedlings under Heat Stress

**Figure 6:**
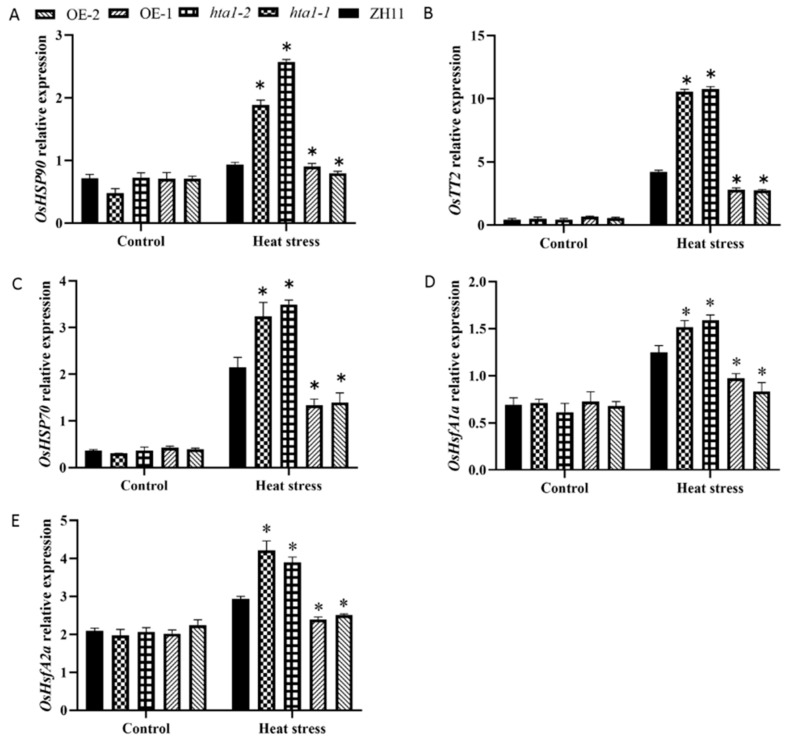
Transcriptional expression of heat-responsive genes (**A**–**E**) in WT, *hat1-1*, *hat1-2*, OE-1, and OE-2 plants under control and heat stress. The data are presented as mean ± standard deviation (*n* = 3; * p < 0.05).

In plants, heat shock proteins (HSPs) are often considered essential for plant survival under heat stress due to their role as molecular chaperones that help maintain and/or restore protein homeostasis [35,36]. Heat stress transcription factors (HSFs) are the most important component in the complex transcriptional regulatory network of plant thermal response, which can trigger a series of complex transcriptional cascades, thereby activating the expression of downstream genes such as transcription factors, active oxygen scavenging enzymes, and HSPs induced by heat response [37]. They can also assist in the refolding of proteins under abiotic stress conditions, restore cellular homeostasis by rebuilding normal protein conformations, and play an important role in protecting plants from abiotic stress [38]. The *OsTT2* protein helps plants reduce energy loss under heat conditions and mitigate the negative impact of heat on plants [39]. Therefore, changes in the expression levels of HSP genes, *OsTT2,* and HSFs in *HTA1* transgenic and WT seedlings were examined using RT-qPCR. Under heat stress, the expression levels of *OsHSP70*, *OsHSP90*, *OsTT2*, *OsHsfA1a*, and *OsHsfA2a* were significantly higher in the mutant strain than those in the WT, while the expression levels of heat-responsive genes in the overexpression lines were significantly lower than those in the WT (Figure 6A–E). Under control conditions, the expression levels of these heat-responsive genes did not differ among treatment lines (Figure 6A–E). These results, combined with changes in the transcript levels of antioxidant- and defense-related genes, suggest that the reduced heat resistance of the *HAT1* overexpression lines may be due to the suppression of the expression of antioxidant- and defense-related genes and heat-responsive genes.

## 4. Discussion

Rice is a warmth-loving crop that originated in tropical and subtropical regions. Temperatures above or below the optimum temperature for rice growth and development are detrimental to the normal growth of rice. With global warming, frequent extreme high temperatures seriously jeopardize the ability of rice to grow normally and maintain yield, and heat stress has become one of the key abiotic factors inhibiting plant growth and crop yield [40]. Therefore, identification of genes which regulate heat stress in plants is extremely important for breeding heat-tolerant rice varieties. The temperature ranges required for the different growth and development stages of rice are different. Heat stress during the seedling stage of rice can lead to stunting of seedling growth, wilting and yellowing of leaves, and even the death of seedlings [41,42]. Our current study shows that *hta1* mutant seedlings exhibit enhanced heat tolerance, whereas seedlings that overexpress *HTA1* exhibit reduced heat tolerance. These findings suggest that *HTA1* plays a negative regulatory role in conferring heat stress tolerance to rice seedlings.

Our results showed that the expression of *HTA1* in ZH11 seedlings was higher than that in control conditions during the first 24 h after heat stress, suggesting that heat induces the expression of *HTA1* (Figure 1). This may be attributed to the short duration of heat treatment that induced the expression of *HTA1* during the first 24 h. In addition, our results showed that the expression of *HTA1* was significantly lower at 24 h of heat treatment than that at 12 h. We hypothesized that further prolongation of heat treatment might lead to a decrease in *HTA1* gene expression. In support of this, *OsMADS87* negatively regulated heat tolerance, and its expression was strongly induced and peaked before 48 h of high-temperature treatment and was sharply down-regulated at 72 h [43].

Biofilms are important for plant defense against various abiotic stresses, and the loss of their integrity is a major indicator of heat-induced damage [44]. The relative electrolyte leakage rate is a parameter reflecting plant cell membrane permeability under abiotic stress [45]. Malondialdehyde (MDA) is an important indicator reflecting the degree of cell membrane peroxidation and membrane damage under stress conditions [46]. When cell membranes are subjected to heat stress, MDA and the electrolyte leakage rate will increase, cell membrane stability will decrease, and damage will be aggravated [47]. In this study, MDA content and electrolyte leakage rate levels were significantly lower in *hta1* mutants compared with WT plants under heat stress, whereas the opposite was true for *HTA1* overexpression lines (Figure 3A–D). This implies that the *hta1* mutants under heat stress can reduce cell membrane damage by maintaining the relative integrity of the cell membrane.

ROS play a vital role in the regulation of plant response to various stress factors [48,49]. Under normal circumstances, the production and clearance of ROS are in a state of dynamic balance, and appropriate ROS can play physiological roles such as signal transduction and cell function regulation. However, when plant cells are damaged by heat, the production of ROS exceeds the clearance defense system, and the accumulated ROS will damage proteins, lipids, nucleic acids, and other biological macromolecules, resulting in the death of plant cells [50]. ROS levels were reported to increase more rapidly in thermosensitive rice than in thermotolerant rice under the same high-temperature treatment, suggesting that ROS accumulation is directly related to plant tolerance to heat stress [51]. In our study, ROS accumulation levels in *hta1* mutants subjected to heat stress were significantly lower compared to WT plants, whereas the opposite was true for *HTA1* overexpression, which resulted in low survival rates (Figure 3F–H and Figure 4E). This suggests that *HTA1* mediates the accumulation of ROS under heat stress.

Antioxidant enzymes (including SOD, POD, CAT, and APX) play an important role in ROS detoxification, and their activities are positively correlated with plant heat tolerance, which is considered to be an important part of heat stress adaptation [52,53]. In the present study, there was no significant difference in antioxidant enzyme activity between wild-type and transgenic plants under control conditions, and the antioxidant enzyme activity of the *hta1* was significantly higher than that of the WT under heat stress (Figure 3E,G), while the antioxidant enzyme activity of the overexpressed lines was significantly lower than that of the WT (Figure 3F,H). Trends in transcript levels of antioxidant-related genes under heat stress follow the same trend as those of antioxidant enzyme activities (Figure 5D–F). These results suggest that the *HTA1* mutant lines enhance the heat tolerance of seedlings, possibly by increasing the enzyme activity of the antioxidant enzyme system to enhance the ROS capacity and thus mitigate the oxidative damage. Consistent with this paper, tomato plants with RNAi-SlMAPK1 and knockdown of *SlMAPK3* were reported to exhibit stronger heat tolerance through increased antioxidant enzyme activities compared to WT plants [54,55]. These results suggest that increasing the enzymatic activity of the antioxidant enzyme system, which in turn reduces ROS accumulation, is one of the pathways by which the *HTA1* mutant line enhances heat tolerance in seedlings. There is also evidence that overexpression of *CuZnSOD* and *APX* improves heat tolerance in potato [56]. On the contrary, *AXP1* and *CAT2* deletion reduced heat stress tolerance in *Arabidopsis thaliana* [57]. In addition, non-enzymatic antioxidants such as ascorbic acid (vitamin C), glutathione, etc., also play an important role in maintaining ROS homeostasis. Zandalinas et al.’s [58] study showed that the maintenance of favorable glutathione (GSH)/oxidized glutathione (GSSG) ratios is strongly associated with heat stress tolerance in citrus. Therefore, in this study, the alteration of ROS accumulation under heat stress may also be due to a combination of enzymatic and non-enzymatic antioxidant systems, and further studies on non-enzymatic antioxidant systems are needed for the follow-up work [59].

It is well known that HSPs and HSFs, as molecular chaperones, play an important role in enhancing plant heat tolerance by regulating downstream genes for plant heat response [60]. In our study, *hta1* had higher relative expression levels of *HSP70* and *HSP90* than WT plants under heat stress, while the overexpressed lines had lower relative expression levels of *HSP70* and *HSP90* than WT plants (Figure 6A,C). *HsfA1a* and *HsfA2a* are key components in the regulation of heat tolerance in plants [61]. *SlHsfA1a*-overexpressing strains showed significantly increased heat tolerance, whereas repressor strains exhibited heat sensitivity [62]. Overexpression of *AtHsfA2* or *SlHsfA3* in *Arabidopsis* can enhance heat stress tolerance in *Arabidopsis thaliana* [63,64]. In our study, *OsHsfA1a* and *OsHsfA2a* had higher transcript levels in *hta1* plants under heat stress, while the overexpressed lines showed the opposite trend (Figure 6D,E). These results suggest that *HTA1*-mediated heat stress tolerance in rice plants may be associated with increased transcript levels of *HSP* and *HSF* genes.

## 5. Conclusions

This study revealed the effect of the GDSL lipase family gene *HTA1* on the heat tolerance of rice seedlings. Under heat treatment, the survival rate of *HTA1* gene-edited lines was significantly higher than that of the WT, whereas *HTA1* gene overexpression lines were significantly lower than that of the WT. Further studies showed that *HTA1* mediates heat stress tolerance in rice seedlings by regulating membrane damage, ROS accumulation, and antioxidant enzyme activities, as well as the expression of antioxidant enzymes and heat-responsive and defense genes. In summary, *HTA1* negatively regulates heat tolerance in rice seedlings. In our subsequent work, we need to pay more attention to the natural variation in *HTA1* and its regulatory role in heat tolerance at rice maturity in order to better understand the exact role of *HTA1* in plant development and heat stress response and to provide valuable genetic resources for crop breeding.

## Data Availability

The data presented in this study are available on request from the corresponding author. The data are not pub-licly available due to for privacy reasons.

## Supplementary Materials

The following supporting information can be downloaded at: https://www.mdpi.com/article/10.3390/antiox13050592/s1, Table S1: Primer names and sequences.
