# Supplementary material for: GDSL Lipase Gene HTA1 Negatively Regulates Heat Tolerance in Rice Seedlings by Regulating Reactive Oxygen Species Accumulation
Source: Antioxidants (Basel). 2024 May 11;13(5):592. doi: 10.3390/antiox13050592 (PMC11117967; doi:10.3390/antiox13050592)
Supplement: Supplementary file 1 [file antioxidants-13-00592-s001.zip › antioxidants-2935756-supplementary.pdf]

Table S1 Primer name and sequence

Primer name	Sequence (5'-3')	Gene symbol
qPHTA1-F	TGATCTAGGAGCCAAGACGAT	LOC4332910
qPHTA1-R	CACCGAGAAGTCATTCAACCA	
OsActin1-F	GACCTTCAACACCCCTGCTA	LOC4333919
OsActin1-R	GAGTCCAACACAATACCTGTGG	
OsCTAB-F	CCCTCCATGTGCCTGTAGTT	LOC4342124
OsCTAB-R	GTTCGGTTCTCCACAGTCGT	
Fe^{-}-SOD-F	GCCAGACCCCAAAAGTGATA	LOC4340091
Fe^{-}-SOD-R	CTTGATGCCCTGGAACCTTA	
OsAXP1-F	CAGTTCGGAGAGCTTGAGGT	LOC4332474
OsAXP1-R	CCAAGGGTTCTGACCACCTA	
OsSNAC1-F	CACACGTTGCAGCATCGATC	LOC4334553
OsSNAC1-R	CATGGTCCCGTTCTGAGGTG	
OsDREB2A-F	TTCCGCTCCTGACAAACACG	LOC4324418
OsDREB2A-R	GGAATCTCCTCCTTTCATCGTG	
OsLEA-F	CACACCCGTCAGAAATCCTC	LOC543476
OsLEA-R	TCACTTCAAATTCGGTGCAA	
OsHSP90-F	GCCAAGCGTCAAGCAGTGACCAA	LOC4342077
OsHSP90-R	GGTCATCAAAGCGCCGCCCTAT	
OsHSP70-F	TTTGGGCGAAGGTGACACTGCTA	LOC4351208
OsHSP70-R	TGGCAATGGTCCCAAGGTTCTTAAT	
OsTT2-F	AAACAGAGGAGCTTGGACCG	LOC9269602
OsTT2-R	ATGTTTCCGCGCTTTATGCC	
OsHSFA1a-F	AAATGATGTCGTTCCTGGC	LOC4334826
OsHSFA1a-R	ATCCTTCGTTTCTTGCTGC	
OsHSFA2a-F,	TAGTGAAAGTGAAGGTCGAAGA	LOC4334080
OsHSFA2a-R	ATGAGAGTCCCAAACAATAAAG	
